# Skin secretion of Andrias davidianus aqueous extract delays skin aging by suppressing the MAPK pathway

**DOI:** 10.1016/j.gendis.2026.102255

**Published:** 2026-05-28

**Authors:** Runqi Zhou, Jing Yang, Liuting Chen, Siyu Peng, Xing Feng, Ximu Zhang, Jinlin Song

**Affiliations:** a The Affiliated Stomatological Hospital of Chongqing Medical University, Chongqing 401147, China; b Chongqing Key Laboratory of Oral Diseases, Chongqing 401147, China; c Chongqing Municipal Key Laboratory of Oral Biomedical Engineering of Higher Education, Chongqing 401147, China; d Chongqing Municipal Health Commission Key Laboratory of Oral Biomedical Engineering, Chongqing 401147, China

**Keywords:** Anti-aging, Antioxidant, D-Gal, MAPK, SSAD, UVB

## Abstract

Skin aging, a prominent manifestation of organismal senescence, is closely associated with the accumulation of endogenous reactive oxygen species (ROS). This study investigated the anti-skin-aging effects of skin secretion of Andrias davidianus (SSAD) using *in vitro* models of human skin fibroblast (HSF) senescence induced by d-galactose (D-Gal) or ultraviolet B (UVB) irradiation and an *in vivo* murine model of UVB-induced photoaging. Our findings demonstrate that *in vitro*, SSAD aqueous extract pretreatment not only mitigated D-Gal/UVB-induced cell death and senescence by enhancing HSF proliferation and migration but also effectively attenuated intracellular ROS overproduction and DNA damage, while upregulating key antioxidant enzymes, including GPX-1 and SOD-1. *In vivo*, SSAD ameliorated UVB-induced dermal thickening, capillary dilation, and collagen degradation, and upregulated P21 expression. Mechanistically, the anti-aging efficacy of SSAD appears to be mediated by a multi-targeted action that suppresses ROS generation, inhibits MAPK signaling pathway activation, and promotes antioxidant enzyme expression. In conclusion, SSAD emerges as a promising natural agent capable of mitigating skin aging by concurrently targeting oxidative stress and modulating the MAPK pathway.

## Introduction

Aging is increasingly regarded as a fundamental pathological process that not only accompanies chronological advancement but also serves as a key driver and predisposing factor for a multitude of age-related diseases, including chronic kidney disease, diabetes, cardiovascular disorders, and Alzheimer's disease.^1^, ^2^, ^3^, ^4^, ^5^, ^6^, ^7^ Consequently, delving into the core mechanisms of aging and exploring effective intervention strategies has become a focal point in biomedical research.

As the primary interface with the external environment, the skin serves as a portal where hallmarks of aging provide clinically accessible insights into the systemic aging processes of organisms.^8^^,^^9^ At the cellular level, the appearance and accumulation of senescent dermal fibroblasts are considered hallmarks of functional decline in skin tissue.^10^^,^^11^ Skin aging is driven by two primary etiological pathways: intrinsic (chronological) and extrinsic (environmental) aging.^12^, ^13^, ^14^ Intrinsic aging represents an inevitable, genetically programmed process characterized by progressive physiological decline over time.^12^^,^^13^ In contrast, extrinsic aging primarily results from environmental exposures, particularly solar ultraviolet (UV) radiation, with the UV-induced component specifically referred to as photoaging.^14^ Spectrally, the ultraviolet spectrum comprises UVA (315–400 nm), UVB (280–315 nm), and UVC (100–280 nm) wavelengths. While UVC is effectively absorbed by the ozone layer, both UVA and UVB radiation penetrate the atmosphere to reach the Earth's surface.^14^ Although UVB has a shorter wavelength and lower penetrating ability, it has a significantly higher photon energy than UVA, enabling it to inflict more direct damage on skin cells and making it a primary agent in photoaging.^15^^,^^16^ Molecularly, UVB radiation exerts dual damaging effects: it not only induces the generation of excessive reactive oxygen species (ROS), thereby activating complex oxidative stress pathways, but also directly absorbs ROS in nuclear DNA, causing DNA lesions.^17^

It is noteworthy that both intrinsic and extrinsic aging pathways converge on cellular events closely associated with excessive ROS generation.^18^^,^^19^ Excessive ROS acts as a potent secondary messenger that can efficiently trigger the cascade activation of the MAPK signaling pathway, culminating in AP-1 activation. This activation drives the transcriptional upregulation of matrix metalloproteinases (MMPs), which subsequently mediate the systemic degradation of collagen and elastin within the extracellular matrix (ECM).^20^^,^^21^ This progressive breakdown of structural components directly compromises skin integrity, manifesting clinically as reduced elasticity and the formation of wrinkles.^21^ Furthermore, ROS and activated MAPK pathways can synergistically trigger NF-κB activation, a pivotal transcription factor. NF-κB activation dysregulates the expression of key pro-inflammatory mediators (e.g., TNF-α, IL-1β, and IL-6), leading to chronic, low-grade inflammation (inflamm-aging) in the skin, which further exacerbates tissue damage and functional impairment.^22^^,^^23^ Beyond disrupting signaling pathways, ROS can directly damage cellular DNA, causing base damage and strand breaks, thereby initiating the DNA damage response, which ultimately leads to cell cycle arrest, senescence, or programmed cell death.^24^ The skin secretion of Andrias davidianus (SSAD), derived from the unique mucus of the long-lived Chinese giant salamander, is a rich source of bioactive compounds.^25^ Notably, SSAD powder contains growth factors that orchestrate key regenerative processes, facilitating stem cell migration and recruitment to injury sites, stimulating cellular proliferation, and promoting collagen synthesis and secretion, thereby significantly enhancing wound healing.^26^, ^27^, ^28^ Previous studies have confirmed that SSAD powder exhibits robust free-radical scavenging capacity, indicating significant antioxidant activity,^28^ suggesting its potential application in counteracting oxidative stress-driven aging processes.

D-galactose (D-Gal) is a naturally occurring monosaccharide. When accumulated excessively *in vivo*, it can induce pronounced oxidative stress through various mechanisms (e.g., promoting advanced glycation end-product formation, disrupting mitochondrial function), thereby mimicking and accelerating the natural aging process.^29^^,^^30^ Therefore, it is widely used in scientific research to establish aging models in cells or animals.^31^^,^^32^

To mechanistically interrogate the anti-skin-aging potential of SSAD aqueous extract, this study implemented a comprehensive experimental strategy. We established complementary *in vitro* and *in vivo* models: human skin fibroblasts (HSFs) senesced by UVB irradiation and D-Gal exposure, and a murine model of chronic UVB-induced cutaneous photodamage in Kunming (KM) mice. This integrated methodology enables a systematic dissection of the underlying molecular mechanisms (Fig. 1).

**Figure 1:**
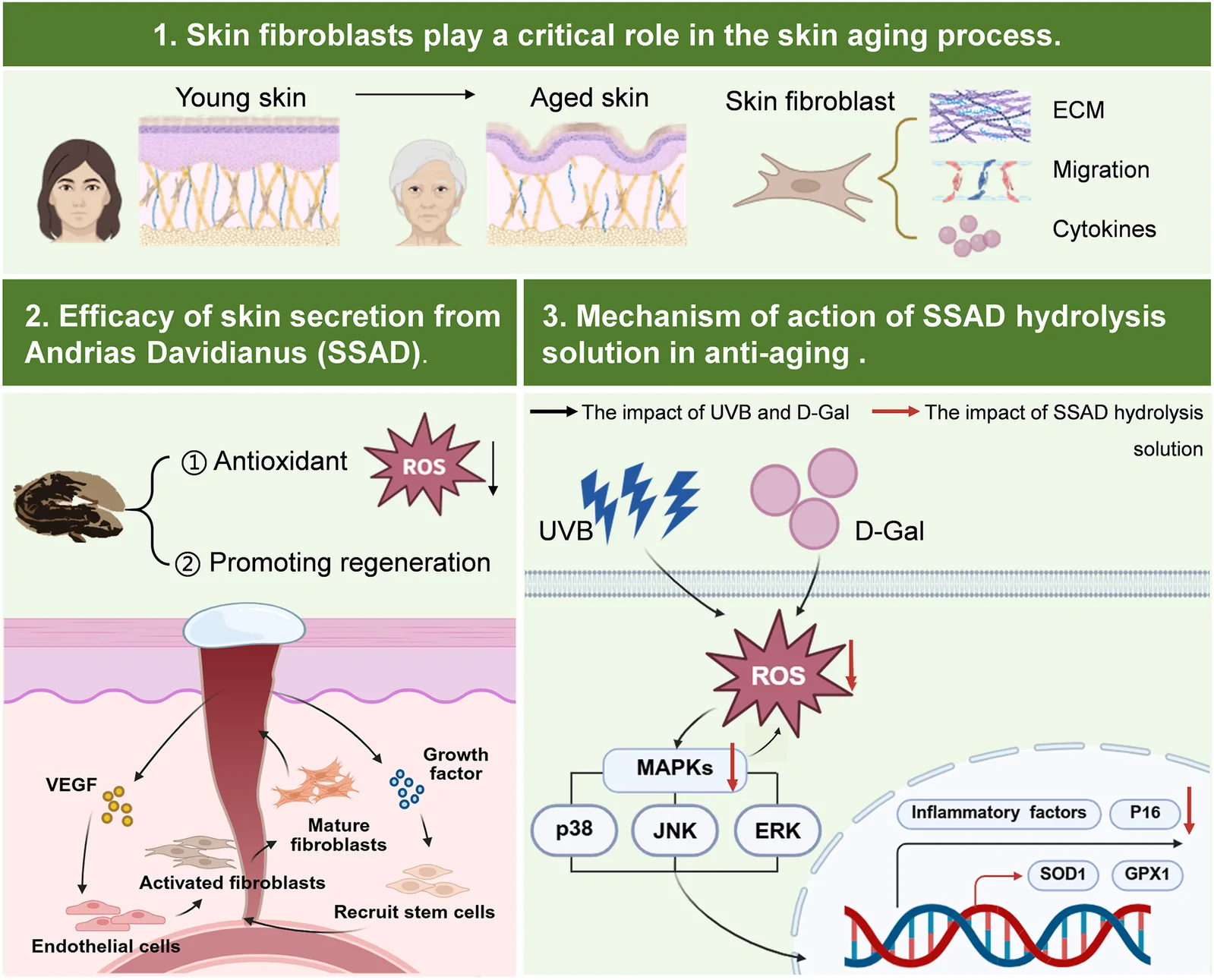
Schematic diagram of the SSAD aqueous extract for skin anti-aging.

## Materials and methods

### Reagents and materials

The SSAD aqueous extract was prepared in our laboratory, while all commercial reagents were obtained from specified suppliers: D-Gal (Sigma–Aldrich, USA); cell culture reagents, including DMEM, penicillin‒streptomycin (Gibco, USA), and fetal bovine serum (FBS) (ExCell Bio, China); assay kits for cell counting kit-8 (CCK-8), ROS detection, RIPA lysis buffer, and bicinchoninic acid (BCA) protein quantification (Beyotime Institute of Biotechnology, China); senescence-associated β-galactosidase (SA-β-Gal) staining kit (Solarbio, China); Western blot antibodies and reagents (Cell Signaling Technology, USA); dihydroethidium (DHE) (Apex Bio, USA); Beyo3D™ Reactive Oxygen Species Assay Kit with 2′,7′-dichlorodihydrofluorescein diacetate (DCFH-DA), horseradish peroxidase (HRP)-labeled goat anti-rabbit IgG, HRP-labeled goat anti-mouse IgG, Alexa Fluor 488-labeled goat anti-rabbit IgG and Alexa Fluor 647-labeled goat anti-mouse IgG (Beyotime, China); Pepsin antigen retrieval solution, 2,2-diphenyl-1-picrylhydrazyl (DPPH) free radical scavenging capacity assay kit and mounting medium, antifading (with 4′,6-diamidino-2-phenylindole (DAPI)) (Solarbio, China); Tissue-Tek O.C.T. compound (SAKURA, USA); and HiScript Q RT SuperMix for quantitative polymerase chain reaction (qPCR) and ChamQ Universal SYBR qPCR Master Mix (Vazyme, China).

### SSAD aqueous extract preparation

SSAD lyophilized powder was mixed with l-ascorbic acid (ASC) powder (acting as a reducing agent) and dissolved in double-distilled water. The mixture was incubated at 4 °C for 48 h and centrifuged, and the supernatant was collected. The supernatant was then dialyzed for 48 h at 4 °C against double-distilled water. The final aqueous extract (termed SSAD aqueous extract) was stored at −80 °C.

### Quantification of ASC content by high-performance liquid chromatography

The concentration of ASC in the samples was determined using a Shimadzu LC-20AD high-performance liquid chromatography system. ASC standard solutions at varying concentrations were prepared and analyzed to generate a standard curve, establishing the linear relationship between response values (peak area) and ASC concentration. The supernatant prior to dialysis and the SSAD aqueous extract after dialysis were collected and analyzed by HPLC under identical conditions. The response values of the samples were measured and substituted into the ASC standard curve to calculate the corresponding ASC concentrations before and after dialysis.

### DPPH radical scavenging assay

The *in vitro* antioxidant capacity of the SSAD aqueous extract was evaluated using the DPPH radical scavenging assay. Briefly, a 0.05 mg/mL DPPH (Solarbio, China) solution was prepared with anhydrous ethanol. The SSAD aqueous extract was mixed at different concentrations with DPPH solution in a 1:9 ratio. After 20 min, the absorbance of the reaction mixture at 515 nm was measured by using a UV–visible spectrophotometer (UV2500, Tianmei, China). The scavenging activity was calculated from absorbance values, and the half-maximal inhibitory concentration (IC50) for both SSAD aqueous extract and ASC (1 mg/mL) was determined by nonlinear regression.

### *In vitro* experiments

#### *Cell culture*

HSFs were isolated from foreskin tissue procured from the Stomatology Hospital of Chongqing Medical University (male, aged 8–10 years). The institutional ethics committee approved the study protocol (CQHS-REC-2024005). Cells were routinely cultured in DMEM supplemented with 10% FBS and 1% penicillin‒streptomycin at 37 °C in a 5% CO_2_ humidified atmosphere. All experiments were conducted using cells between passages 3 and 8 during the logarithmic growth phase.

#### *Establishment of HSF senescence models*

To simulate *in vivo* aging environments, both a D-Gal-induced replicative senescence model and a UVB-induced photoaging model were established. First, to determine the optimal induction conditions, HSFs were seeded at a density of 5 × 10^4^ cells/mL. After 24 h of adhesion, cells were exposed to various concentrations of D-Gal (10, 20, 40, 80 mg/mL) or different doses of UVB radiation (150, 180, 200, 250 mJ/cm^2^). Subsequent cell viability assays were performed to select appropriate modeling parameters. For the D-Gal-induced senescence experiment, the following groups were set: 1) Control group: cultured in standard complete medium; 2) D-Gal group: treated with conditional medium containing 40 mg/mL D-Gal; 3) SSAD + D-Gal group: pretreated with medium containing 0.005% SSAD aqueous extract (20 μg/mL) for 24 h, followed by co-treatment with conditional medium containing both 40 mg/mL D-Gal and 0.005% SSAD aqueous extract; 4) ASC + D-Gal group: pretreated with medium containing 0.1 mg/mL ASC solution for 24 h, followed by co-treatment with conditional medium containing both 40 mg/mL D-Gal and 0.1 mg/mL ASC solution.

For the UVB-induced photoaging experiment, the groups were as follows: 1) Control group: routine culture without UVB irradiation; 2) UVB group: irradiated with UVB at a dose of 200 mJ/cm^2^; 3) SSAD + UVB group: pretreated with medium containing 0.005% SSAD aqueous extract for 24 h and then irradiated with UVB at 200 mJ/cm^2^ alongside the UVB group; and 4) ASC + UVB group: pretreated with medium containing 0.1 mg/mL ASC solution for 24 h and then irradiated with UVB at 200 mJ/cm^2^ alongside the UVB group. During irradiation, all irradiated groups were covered with a thin layer of PBS to ensure uniform exposure.

#### *UVB irradiation protocol*

In the treatment of HSF cells mentioned above, a layer of PBS was applied to cover the cells during UVB irradiation (200 mJ/cm^2^). Cell irradiation was performed using a custom-made irradiation box equipped with three UVB lamps (G20T8E; wavelength range: 280–320 nm, peak: 313 nm; YIXIAN, China). Following irradiation, PBS was replaced with the corresponding conditional medium to culture the cells.

#### *Cell viability assay*

Cell viability was assessed using the CCK-8 assay. At 24, 48, and 72 h post-treatment, the CCK-8 working solution was added to the cell culture plates, followed by incubation at 37 °C for 2 h. The absorbance (OD) was then measured at 450 nm using a microplate reader (EnSpire, PerkinElmer, USA).

#### *Intracellular ROS level detection*

Intracellular ROS levels were quantified fluorometrically using the cell-permeable probe DCFH-DA. Following experimental treatments, cells were incubated with 10 μM DCFH-DA in serum-free medium at 37 °C for 30 min in the dark. After thorough washing with PBS to remove the extracellular probe, fluorescence images were captured with an EVOS cell imaging system (EVOS M5000, Thermo, USA), and the mean fluorescence intensity was analyzed using ImageJ to determine relative ROS levels.

#### *SA-β-gal staining*

HSFs were treated as previously outlined. At the 48-h time point after stimulation, SA-β-Gal staining was performed to estimate the number of aged cells using a histochemical staining kit. Images were collected, and SA-β-Gal-positive cells were analyzed.

#### *Cell migration assay*

The impact of SSAD on HSF migration was assessed using a scratch wound assay and a transwell migration assay. A scratch was created by using a sterile pipette tip when HSFs were stimulated by UVB/D-Gal in the scratch wound assay. After 24 h of incubation in the corresponding conditional medium, HSF migration toward the scratched area was observed. For the transwell migration assay, stimulated HSFs were seeded in the upper chambers of transwell plates with serum-free DMEM. The lower chamber contained the same conditional medium as described above, and the cells were cultured for 24 h to induce migration. Migrated HSFs were fixed with 4% paraformaldehyde and stained with 0.5% crystal violet (Sigma, USA) for visualization.

#### *Immunofluorescence (IF) staining*

Following experimental treatments, cells on coverslips underwent standard immunofluorescence processing: fixation with pre-cooled methanol (4 °C, 20 min), permeabilization with 0.1% Triton X-100 (room temperature (RT), 15 min), and blocking with 1% BSA/0.5% Tween-20 in PBS (RT, 1 h). Subsequent incubations included primary antibodies (4 °C, overnight) and corresponding fluorophore-conjugated secondary antibodies (RT, 2 h, in the dark), with thorough PBS washes between each step. Finally, the nuclei were counterstained with DAPI (5 min) before being mounted and imaged by fluorescence microscopy (antibodies provided in Table S2).

#### *RNA sequencing and data processing*

Total RNA was isolated from differentially treated HSFs using TRIzol reagent, followed by rigorous quality assessment of concentration, purity, and integrity using NanoDrop spectrophotometry and an Agilent 2100 Bioanalyzer. Qualified RNA samples underwent library preparation with the Illumina Stranded mRNA Prep Kit (including mRNA enrichment, fragmentation, cDNA synthesis, end repair/A-tailing, adapter ligation, and PCR amplification) and subsequent paired-end sequencing (PE150) on an Illumina NovaSeq 6000 platform at Majorbio Biotech Co., Ltd. (Shanghai, China). Raw sequencing reads were processed through fastp for quality control and adapter trimming, followed by alignment to the human reference genome (GRCh38) using HISAT2. Finally, featureCounts was employed to generate the gene expression matrix by assigning aligned reads to genomic features.

#### *Western blot analysis*

RIPA lysis buffer was used to extract proteins from different treatment groups. The protein concentration in the samples was determined using the BCA Protein Assay Kit. After electrophoresis, transfer, and incubation with primary and secondary antibodies (antibodies provided in Table S2), β-actin was used as the internal control. Finally, the target protein levels were detected by a ChemiDoc™ Imaging System (Bio-Rad, USA).

#### *Gene set variation analysis (GSVA)*

To explore the potential mechanisms of the SSAD aqueous extract's anti-aging effects at the pathway level, GSVA was performed. Based on the gene expression matrix, the GSVA R package was used to transform gene-level expression changes into enrichment scores for predefined gene sets (e.g., KEGG and hallmark pathways). By comparing the GSVA scores of specific pathways (e.g., cellular senescence, oxidative stress, DNA damage response) between different treatment groups, significantly regulated biological pathways were identified using thresholds of |GSVA score fold change| > 1 and an adjusted *p*-value < 0.05.^33^, ^34^, ^35^, ^36^, ^37^, ^38^, ^39^, ^40^, ^41^, ^42^

#### *Quantitative real-time PCR (qRT-PCR) analysis*

Gene expression analysis was performed by qRT-PCR following total RNA extraction from HSFs with the RNA-easy™ Isolation Reagent. After quantifying RNA concentration and purity using a Nanodrop ND-2000 spectrophotometer, 1 μg of total RNA was reverse-transcribed into cDNA. Amplification reactions were conducted on a QuantStudio Real-Time PCR System using SYBR Green Premix under the following thermal cycling conditions: initial denaturation at 95 °C for 30 s, followed by 40 cycles of 95 °C for 5 s and 60 °C for 30 s. Relative gene expression was normalized to GAPDH and calculated using the 2^−ΔΔCt^ method, with all primer sequences provided in Supplementary Table S1.

### *In vivo* experiments

#### *Skin UVB photoaging model establishment*

All animal procedures were approved by the Institutional Animal Care and Use Committee (CQHS-REC-2023188) and conducted in accordance with the NIH Guide for the Care and Use of Laboratory Animals. KM mice (6 weeks old, weighing 18–20 g) were acclimatized for one week in an SPF facility prior to experimentation.

Mice were randomly allocated into three groups (*n* = 8): 1) Control group: the back skin of mice was treated with a wet compress of physiological saline for 30 min daily without UVB irradiation. 2) UVB model group: The back skin was treated with a physiological saline compress for 30 min, followed by UVB irradiation. 3) UVB + SSAD treatment group: The back skin was treated with a wet compress of 0.005% SSAD aqueous extract (20 μg/mL) for 30 min, gently wiped with double-distilled water, and then subjected to UVB irradiation at the same dose as the model group. Prior to the experiment, the hair on a 3 × 3 cm area was shaved. Before each irradiation session, the mice were anesthetized with tribromoethanol and placed in a custom-made restraint box. The shaved dorsal skin was uniformly irradiated at close range using a UVB lamp (power density 1 mW/cm^2^). The initial irradiation lasted 40 min, followed by 1 h of daily irradiation for 7 consecutive days, resulting in a cumulative UVB dose of approximately 24 J/cm^2^. On day 8 after the final irradiation, the mice were euthanized, and dorsal skin samples were collected.

### *In vivo* efficacy study in KM mice

Following 7 days of UVB irradiation, skin physiological parameters were assessed using a DermaScan® C USB skin physiology analyzer (Cortex Technology, Denmark). The measured parameters included dermal collagen levels, skin thickness, and transepidermal water loss (TEWL).

### *In vivo* ROS measurement

Mice were intravenously injected with DHE (10 mg/kg) 24 h prior to sacrifice. Dorsal skin tissues were collected and fixed in 4% paraformaldehyde in the dark for 14 h, followed by gradient dehydration in 10%, 15%, and 30% sucrose solutions. Tissues were then embedded in OCT compound and cryosectioned at 10 μm thickness under light-protected conditions. Sections were hydrated with PBS for 5 min, permeabilized with 0.3% Triton X-100 in the dark for 10 min, and mounted with DAPI-containing anti-fade mounting medium. Fluorescence images were captured for subsequent analysis.

### Histological evaluation

Formalin-fixed skin tissues were processed through dehydration, paraffin embedding, and sectioning (7 μm thickness) for comprehensive histological evaluation. Consecutive sections underwent either hematoxylin and eosin (H&E) or Van Gieson (VG) staining following standard deparaffinization and rehydration protocols. H&E-stained sections enabled assessment of epidermal thickness (quantified using ImageJ), dermal architecture, and inflammatory cell infiltration. VG staining specifically highlighted collagen fibers (pink) for semi-quantitative analysis of dermal collagen area and density. For immunofluorescence staining, sections were deparaffinized, rehydrated, and subjected to pepsin antigen retrieval for 20 min. After blocking with 5% goat serum for 30 min, the slides were incubated overnight at 4 °C with primary antibodies against P21 (1:200), collagen I (1:200), and CD31 (1:200). Following rewarming, sections were incubated with Alexa Fluor 488- or 594-conjugated secondary antibodies (1:500) for 1 h in the dark. Nuclei were counterstained with DAPI-containing mounting medium. Fluorescence images were captured and analyzed.

### Statistical analysis

All quantitative data are expressed as the mean ± standard deviation. Statistical comparisons were performed using one-way ANOVA with Tukey's post hoc test for homogeneous variances in GraphPad Prism 9, with *p* < 0.05 considered statistically significant.

## Results

### Scavenging ability and cytotoxicity of SSAD aqueous extract

Different experimental groups were processed as depicted in Figure 2A. First, SSAD powder was prepared as an SSAD aqueous extract (Fig. S1). High-performance liquid chromatography analysis revealed that the prepared SSAD contained almost no ascorbic acid, thereby ruling out potential effects of ascorbic acid and ensuring that subsequent experimental results were attributable solely to SSAD (Fig. S2; Table S3). The DPPH assay is commonly used to assess the free radical-scavenging activity of antioxidants. The SSAD aqueous extract exhibited DPPH radical-scavenging activity with an IC_50_ value of 4.56 mg/mL in the DPPH assay (Fig. 2B). The SSAD aqueous extract demonstrated the potential to promote cell proliferation (Fig. S3). UVB irradiation and D-Gal are known detrimental factors that induce cell aging and even cell death. The protective effects of SSAD against UVB-induced cell death and D-Gal-induced cell death were assessed using the CCK-8 assay (Fig. 2C and D). In the photoaging model, at a UVB exposure dose of 200 mJ/cm^2^, UVB exposure led to a 28.2% decrease in HSF cell viability after 24 h compared to the control group. However, SSAD treatment demonstrated a time-dependent recovery in cell viability (Fig. 2E). In the D-Gal model, D-Gal significantly induced 18.2% cell death at 48 h compared to the control group; the SSAD group (D-Gal + SSAD) performed even better than the control group (Fig. 2F). These results indicate that SSAD exhibits antioxidant capacity and can protect cells against senescence.

**Figure 2:**
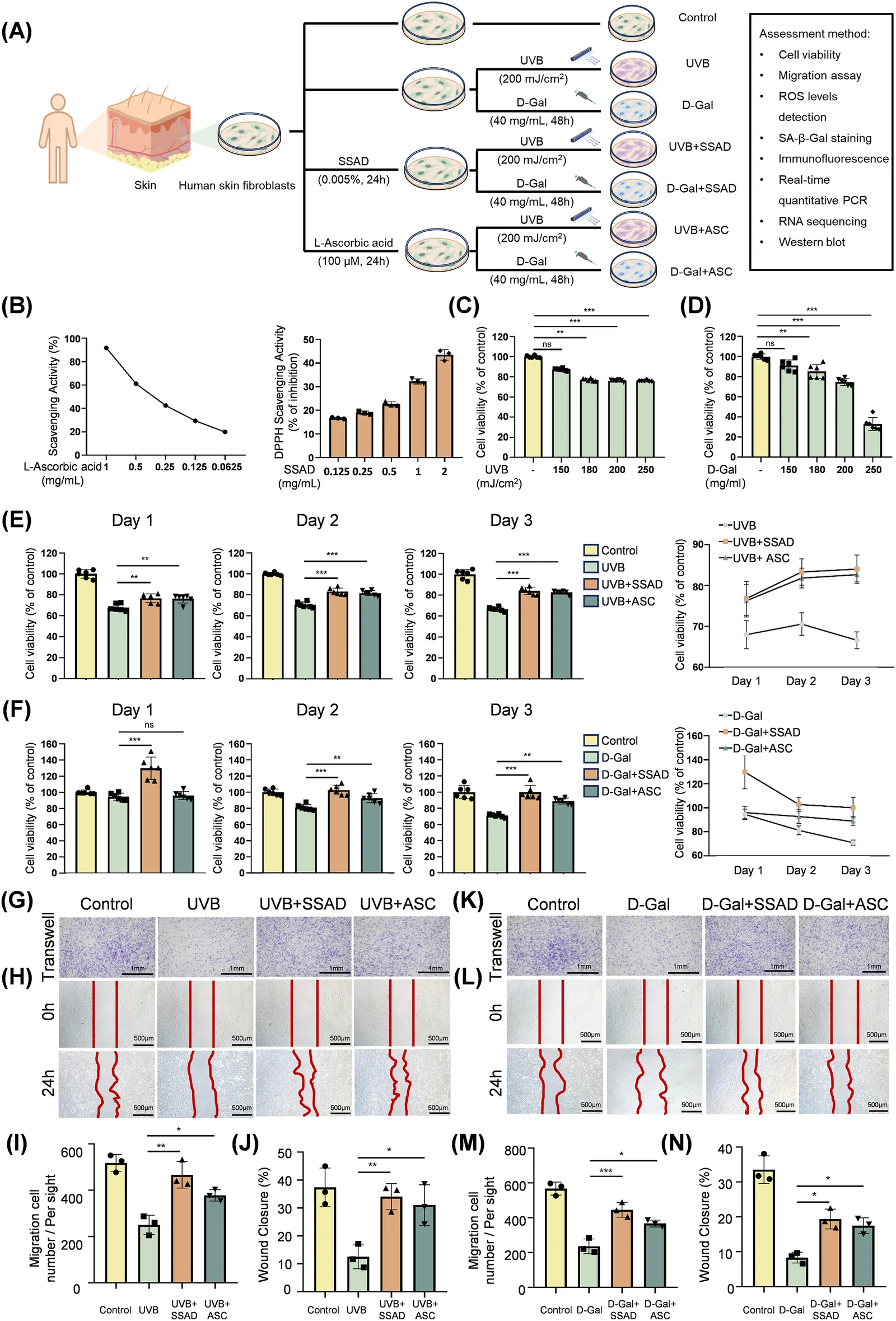
Free radical scavenging ability, cytotoxicity assessment, and cell migration evaluation of SSAD aqueous extract. **(A)** Schematic diagram of the experimental grouping and workflow *in vitro*, outlining the treatment procedures for SSAD aqueous extract in both UVB-induced photoaging and D-Gal-induced replicative senescence models. **(B)** DPPH radical scavenging assay evaluating the *in vitro* antioxidant capacity of SSAD aqueous extract (*n* = 3). ASC served as the positive control. **(C, D)** Effect of different doses of (C) UVB irradiation and (D) D-Gal on the viability of HSFs (*n* = 5), used to determine the optimal conditions for subsequent model establishment. **(E, F)** Effect of SSAD aqueous extract on the viability of (E) UVB-induced photoaged HSFs and (F) D-Gal-induced senescent HSFs (*n* = 6). **(G)** Representative images of Transwell migration assays for UVB-induced senescent HSFs. **(H)** Representative scratch wound images taken at the indicated time points (0 h, 24 h) for the UVB model. **(I)** Quantitative analysis of Transwell migration assays for the UVB model. **(J)** Quantitative analysis of scratch wound assays for the UVB model. **(K)** Representative images of Transwell migration assays for D-Gal-induced senescent HSFs. **(L)** Representative scratch wound images taken at the indicated time points (0 h, 24 h) for the D-Gal model. **(M)** Quantitative analysis of Transwell migration assays for the D-Gal model. **(N)** Quantitative analysis of scratch wound assays for the D-Gal model. In all data, compared to the damage model group, ∗*p* < 0.05, ∗∗*p* < 0.001, ∗∗∗*p* < 0.0001.

### SSAD improves the migration ability of HSFs

In the Transwell migration experiment, the migration ability of HSFs was compromised by UVB or D-Gal treatment. However, SSAD or ASC demonstrated the ability to ameliorate the decline in cell migration after stimulation (Fig. 2G–I, K, M). The cell scratch test results showed a significant decrease in cell migration to the scratch area upon exposure to UVB irradiation or D-Gal treatment, signifying a significant reduction in cell migration ability following stimulation. Notably, in both experimental models, the number of cells moving toward the scratch area increased significantly when SSAD or ASC was administered compared with the UVB and D-Gal groups (Fig. 2H–J, L, N). Moreover, it is worth noting that HSF migration was best restored after SSAD treatment.

### SSAD delays the aging of HSF cells

The SA-β-gal histochemical staining assay was used to examine SA-β-gal, a cellular senescence biomarker, in HSFs. Increased activity of the β-galactosidase enzyme is observed in aging cells, which can be visualized as blue staining in SA-β-gal staining. The results revealed that UVB radiation and D-Gal significantly increased the ratio of positively stained cells. However, pretreatment with SSAD or ASC prior to UVB exposure and D-Gal treatment significantly inhibited the increase in SA-β-gal activity (Fig. 3A, B, E, F). γ-H2AX is a core biomarker of cellular senescence. The level of γ-H2AX increased significantly in the UVB and D-Gal groups and notably decreased in the SSAD and ASC groups (Fig. 3I−L). Through the detection of genes related to aging (such as P16) and inflammation (such as IL-1β), SSAD and ASC treatment alleviated cellular senescence (Fig. 3M and N).

**Figure 3:**
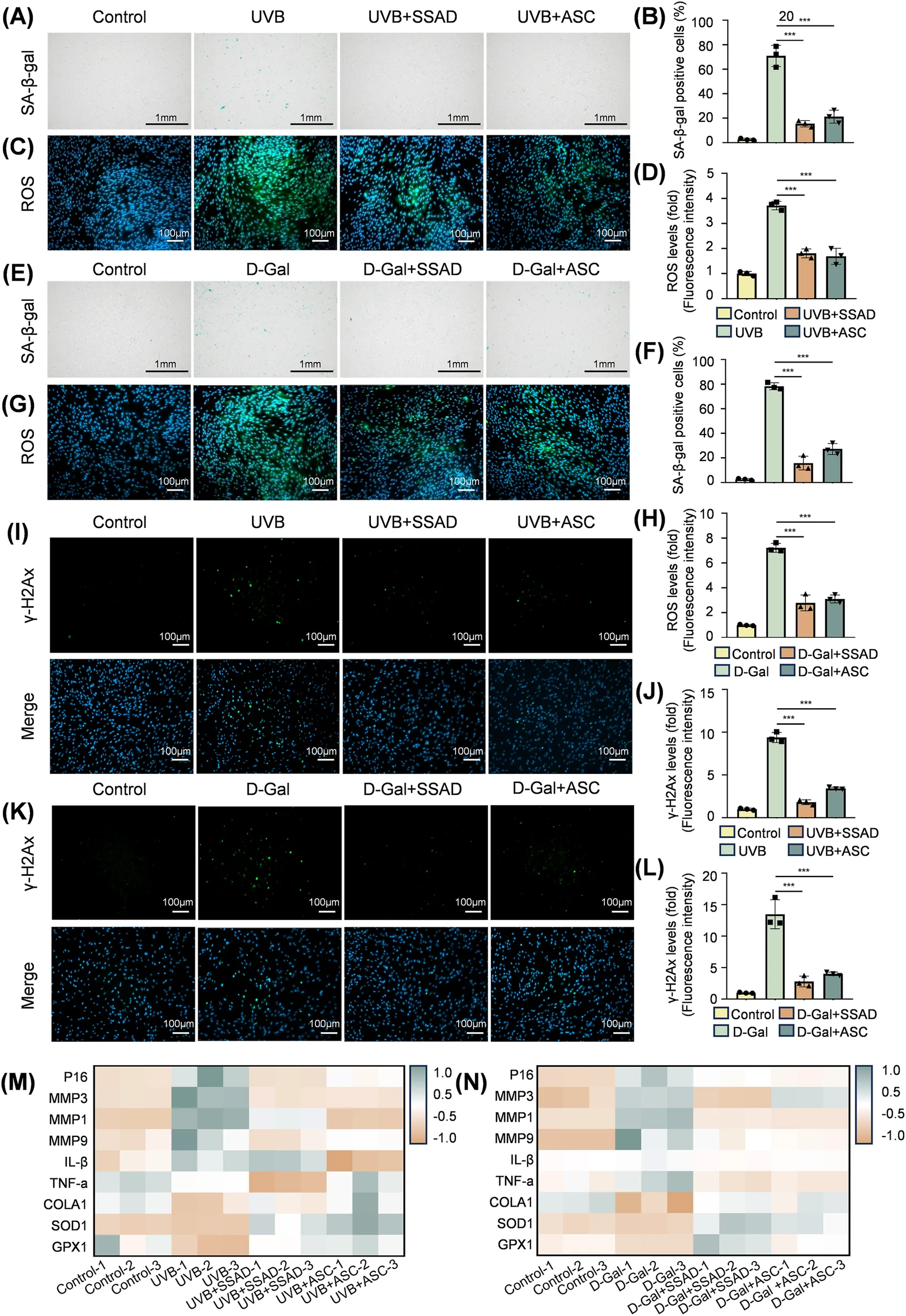
SSAD aqueous extract delays HSF senescence and enhances intracellular antioxidant capacity. **(A)** Representative SA-β-gal staining images of UVB-induced senescent HSFs. **(B)** Quantitative analysis of the percentage of SA-β-gal-positive cells. **(C)** Representative fluorescence images of intracellular ROS levels in the UVB model. **(D)** Quantitative analysis of ROS fluorescence intensity. **(E)** Representative SA-β-gal staining images of D-Gal-induced senescent HSFs. **(F)** Quantitative analysis of the percentage of SA-β-gal-positive cells. **(G)** Representative fluorescence images of intracellular ROS levels in the D-Gal model. **(H)** Quantitative analysis of ROS fluorescence intensity. **(I)** Representative IF staining images for the DNA damage marker γ-H2AX (green) and nuclear counterstain DAPI (blue) in the UVB model. **(J)** Quantitative analysis of γ-H2AX fluorescence intensity. **(K)** Representative IF staining images for γ-H2AX (green) and DAPI (blue) in the D-Gal model. **(L)** Quantitative analysis of γ-H2AX fluorescence intensity. qRT-PCR detection of the transcriptional levels of genes related to senescence and antioxidant response (e.g., p16, MMP1, MMP3, SOD1, GPX1) in the **(M)** UVB model and **(N)** D-Gal model (*n* = 3). ∗∗∗*p* < 0.0001.

### SSAD enhances intracellular antioxidant capacity

Aging cells often exhibit increased ROS generation and accumulation. The control group displayed weak fluorescence, whereas the UVB and D-Gal groups exhibited strong fluorescence, indicating higher ROS levels. The SSAD aqueous extract group demonstrated significantly lower fluorescence intensity than the control group. Antioxidant enzymes play a crucial role in clearing excess ROS and enhancing cell resistance to oxidative damage (Fig. 3C, D, G, H). The qRT-PCR results demonstrated that SSAD aqueous extract increased the mRNA expression of the antioxidant enzymes SOD1 and GPX1 (Fig. 3M and N). These findings indicate that SSAD can reduce ROS levels and enhance the expression of antioxidant enzymes in cells exposed to aging models. According to these results, ASC had a more pronounced effect on ROS removal in the UVB model, while the impact of SSAD and ASC was similar in the D-Gal treatment group.

### Effect of SSAD on MAPK signaling

GSVA analysis results revealed that in both the D-Gal and UVB models, the MAPK pathway was downregulated in the SSAD-treated groups (Fig. 4A and B). Subsequently, we examined the expression levels of key molecules in the MAPK pathway. qRT-PCR results demonstrated that both D-Gal and UVB stimulation significantly upregulated the mRNA expression of MAPK pathway-related genes (e.g., JNK, ERK, P38) compared with their respective controls (Fig. S4). Notably, at the protein level, Western blot analysis showed that treatment with 0.005% SSAD aqueous extract (20 μg/mL) attenuated MAPK pathway activation, as evidenced by reduced phosphorylation of JNK, ERK, and p38 in both senescence models (Fig. 4C and D; the original blots are shown in Fig. S5). This suggests that SSAD has the potential to modulate the MAPK signaling pathway, contributing to its impact on cellular responses to D-Gal and UVB-induced aging.

**Figure 4:**
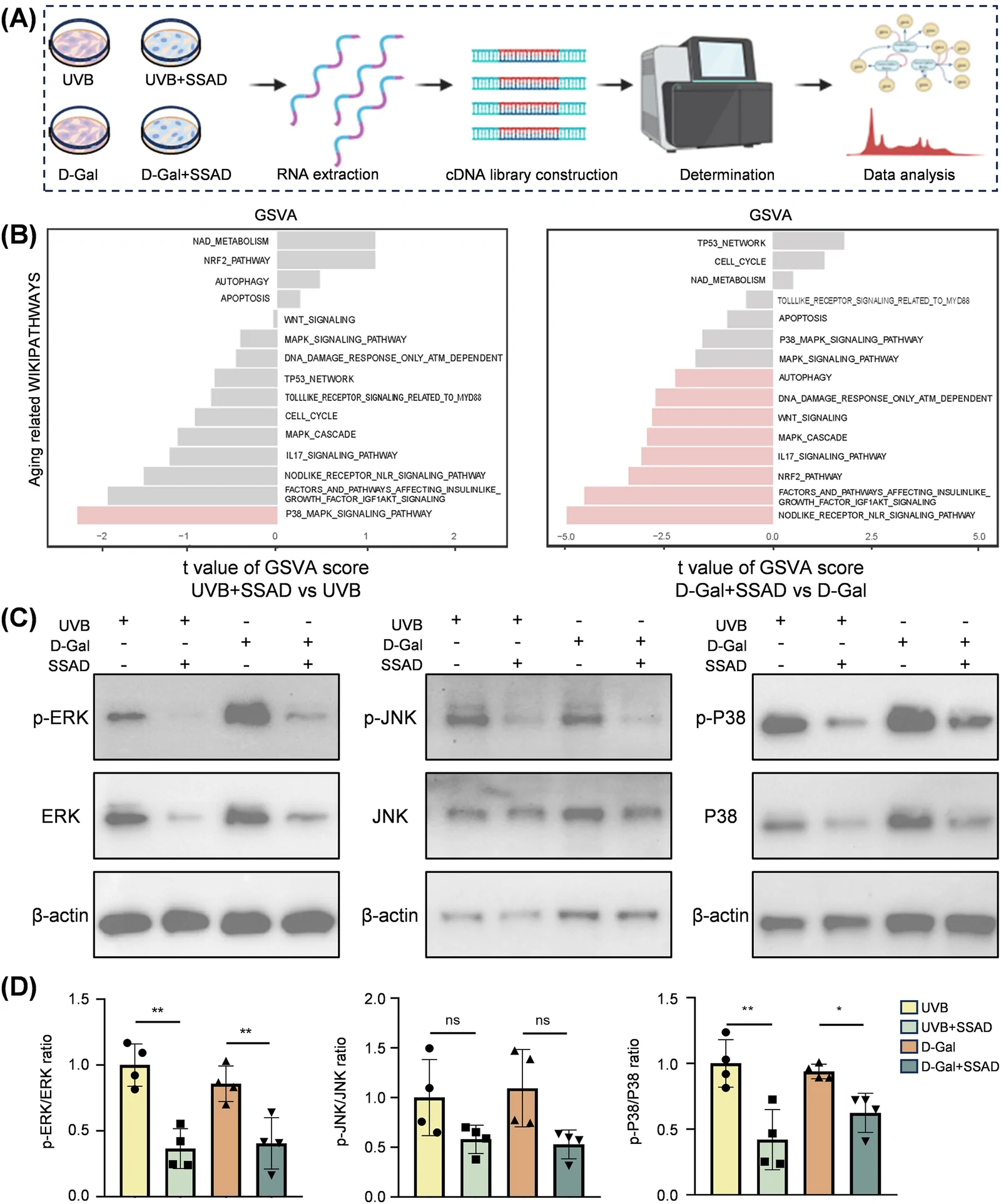
Effect of SSAD aqueous extract on the MAPK signaling pathway. **(A)** Schematic diagram of the transcriptome sequencing experimental procedure. **(B)** GSVA analysis revealing the differential pathways significantly regulated by SSAD aqueous extract in both UVB and D-Gal models, with the MAPK pathway being commonly suppressed. **(C)** Western blot analysis showing the effect of SSAD aqueous extract on the phosphorylation levels of JNK, ERK, and P38 (i.e., p-JNK, p-ERK, p-P38) in HSFs. **(D)** Quantitative analysis of the phosphorylation levels of JNK, ERK, and P38 in HSFs (*n* = 4). ∗*p* < 0.05, ∗∗*p* < 0.001.

### SSAD modulates the ROS-MAPK axis in cellular senescence

To determine the optimal ROS scavenging concentration for subsequent experiments, cells were treated with varying concentrations of NAC in both UVB- and d-galactose-induced senescence models. As shown in Figure 5A and B, NAC scavenged ROS in a dose-dependent manner. Notably, no statistically significant difference in ROS scavenging efficacy was observed between the 5 mM and 10 mM NAC groups, leading to the selection of 5 mM NAC for further studies in both models. Western blot analysis was performed to investigate the regulatory relationship between SSAD, ROS, and the MAPK signaling pathway (Fig. 5C–J). In cells treated with SSAD alone, the p-JNK/JNK ratio decreased to 74.7% and 77.9% of control levels in the UVB and D-Gal models, respectively, suggesting that SSAD may directly suppress JNK phosphorylation. Treatment with NAC alone resulted in no significant changes in the phosphorylation levels of JNK, ERK, or P38, indicating that NAC does not directly modulate these MAPKs under basal conditions. However, in the UVB- or D-Gal-induced senescence models, NAC treatment significantly attenuated the activation of all three MAPK subfamilies. This suggests that ROS mediates the activation of the MAPK pathway under senescence-inducing stress. Furthermore, the downregulation of MAPK phosphorylation by SSAD was more pronounced in the presence of UVB or D-Gal stimulation than with SSAD treatment alone, suggesting that SSAD regulates the MAPK pathway through both direct and ROS-dependent indirect mechanisms. The efficacy of specific MAPK pathway inhibitors (PD98059 for ERK, SP600125 for JNK, and SB203580 for p38) was validated by Western blot (Fig. 5K–P). To further explore the bidirectional crosstalk, cells were pretreated with these inhibitors. Subsequent analysis revealed that inhibition of the MAPK pathway significantly reduced ROS levels (Fig. 5Q and R), indicating that the MAPK cascade can also reciprocally regulate ROS production. Notably, treatment with SSAD demonstrated a superior ROS-scavenging effect compared to any of the three MAPK inhibitors alone.

**Figure 5:**
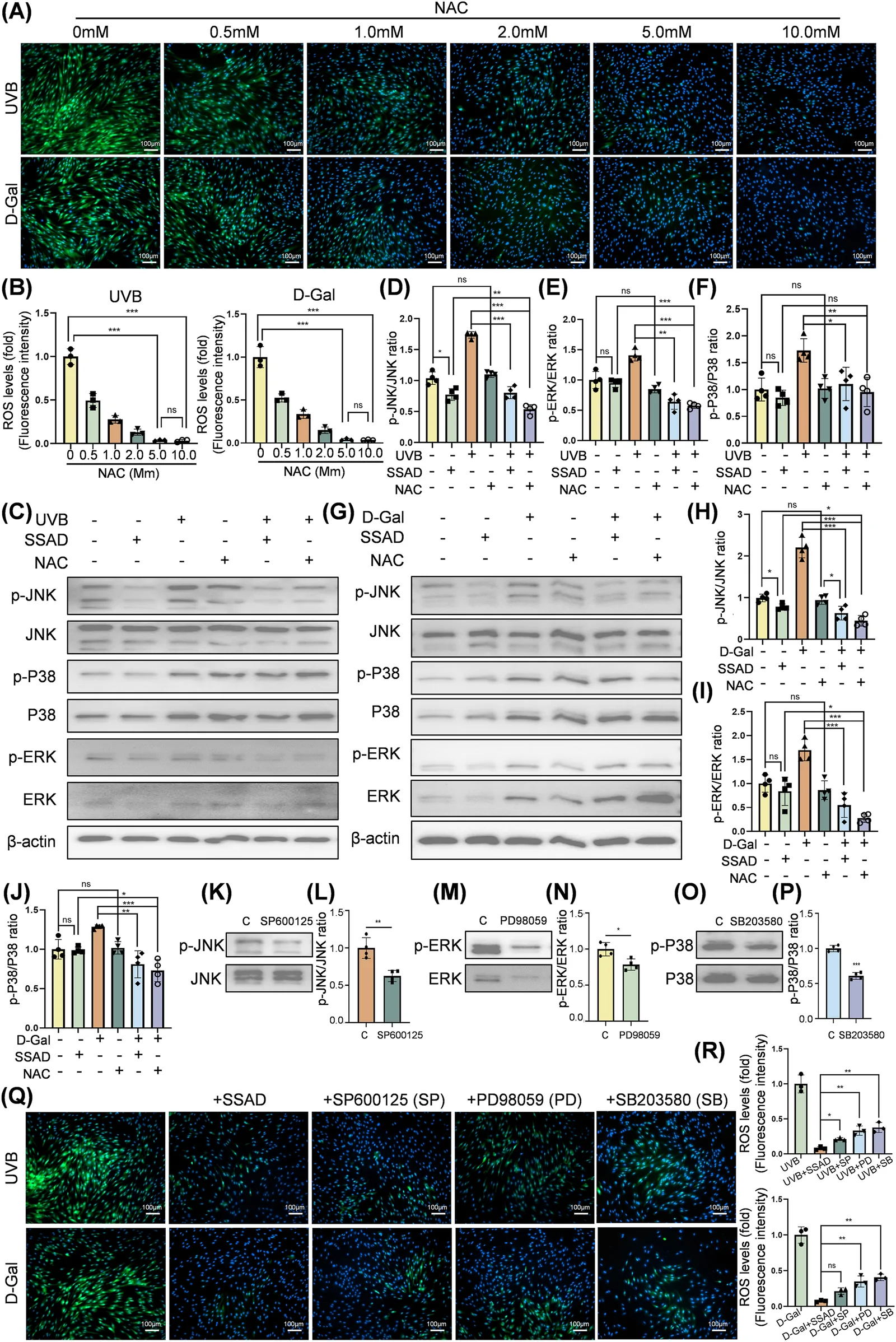
SSAD regulates the MAPK pathway directly and indirectly via ROS, with reciprocal regulation of ROS by MAPK inhibition. **(A)** Representative fluorescence images of ROS levels in UVB- or d-galactose-treated cells following incubation with the indicated concentrations of NAC (0, 0.5, 1.0, 2.0, 5.0, 10.0 mM). **(B)** Quantitative analysis of ROS fluorescence intensity. **(C)** Western blot analysis showing the phosphorylation levels of JNK, ERK, and P38 (i.e., p-JNK, p-ERK, p-P38) in the UVB-photoaging model. **(D**–**F)** Quantitative analysis of the phosphorylation levels of JNK, ERK, and P38 in HSFs (*n* = 4). **(G)** Western blot analysis showing the phosphorylation levels of JNK, ERK, and P38 (i.e., p-JNK, p-ERK, p-P38) in the D-Gal model. **(H**–**J)** Quantitative analysis of the phosphorylation levels of JNK, ERK, and P38 in HSFs (*n* = 4). **(K–P)** HSFs were pretreated with PD98059 (5 μM), SP600125 (10 μM), or SB203580 (5 μM) for 2 h. p-JNK, p-p38, and p-ERK levels were determined by Western blot (*n* = 4). **(Q)** Representative fluorescence images of ROS levels in UVB- or d-galactose-treated cells following incubation with SSAD, PD98059, SP600125, or SB203580. (R) Quantitative analysis of ROS fluorescence intensity (*n* = 3). ∗*p* < 0.05, ∗∗*p* < 0.001,∗∗∗*p* < 0.0001.

### SSAD attenuates skin cell senescence via MAPK pathway modulation

To further elucidate the role of the MAPK pathway in SSAD-mediated protection against cellular senescence, the effects of SSAD and specific MAPK inhibitors on senescence-associated markers were examined. Western blot analysis was performed to assess the expression of p21 (a key senescence marker) and collagen type I (COL1A1), a critical component of the extracellular matrix. As shown in Figure 6A–F, exposure to UVB or d-galactose significantly upregulated p21 expression and downregulated COL1A1 levels. However, treatment with SSAD or MAPK inhibitors (PD98059, SP600125, and SB203580) effectively reversed these changes, with SSAD demonstrating the greatest potency in restoring expression profiles toward basal levels. Consistently, SA-β-gal staining revealed that UVB or d-galactose stimulation markedly increased the proportion of senescent cells. This effect was significantly attenuated by treatment with either SSAD or the MAPK inhibitors, with SSAD again exhibiting the greatest reduction in senescent cell populations (Fig. 6G–J). To investigate the potential mechanisms underlying collagen preservation, the mRNA levels of matrix metalloproteinases (MMPs) were quantified by qRT-PCR. The results demonstrated that SSAD, as well as all three MAPK inhibitors, significantly downregulated the UVB- or d-galactose-induced transcriptional upregulation of MMP1, MMP3, and MMP9 (Fig. 6K and L). This suggests that SSAD may mitigate collagen degradation by suppressing MMP expression through modulation of the MAPK cascade. Collectively, these results demonstrate that SSAD alleviates skin cell senescence through the MAPK signaling pathway, as evidenced by the coordinated regulation of senescence markers and matrix remodeling enzymes.

**Figure 6:**
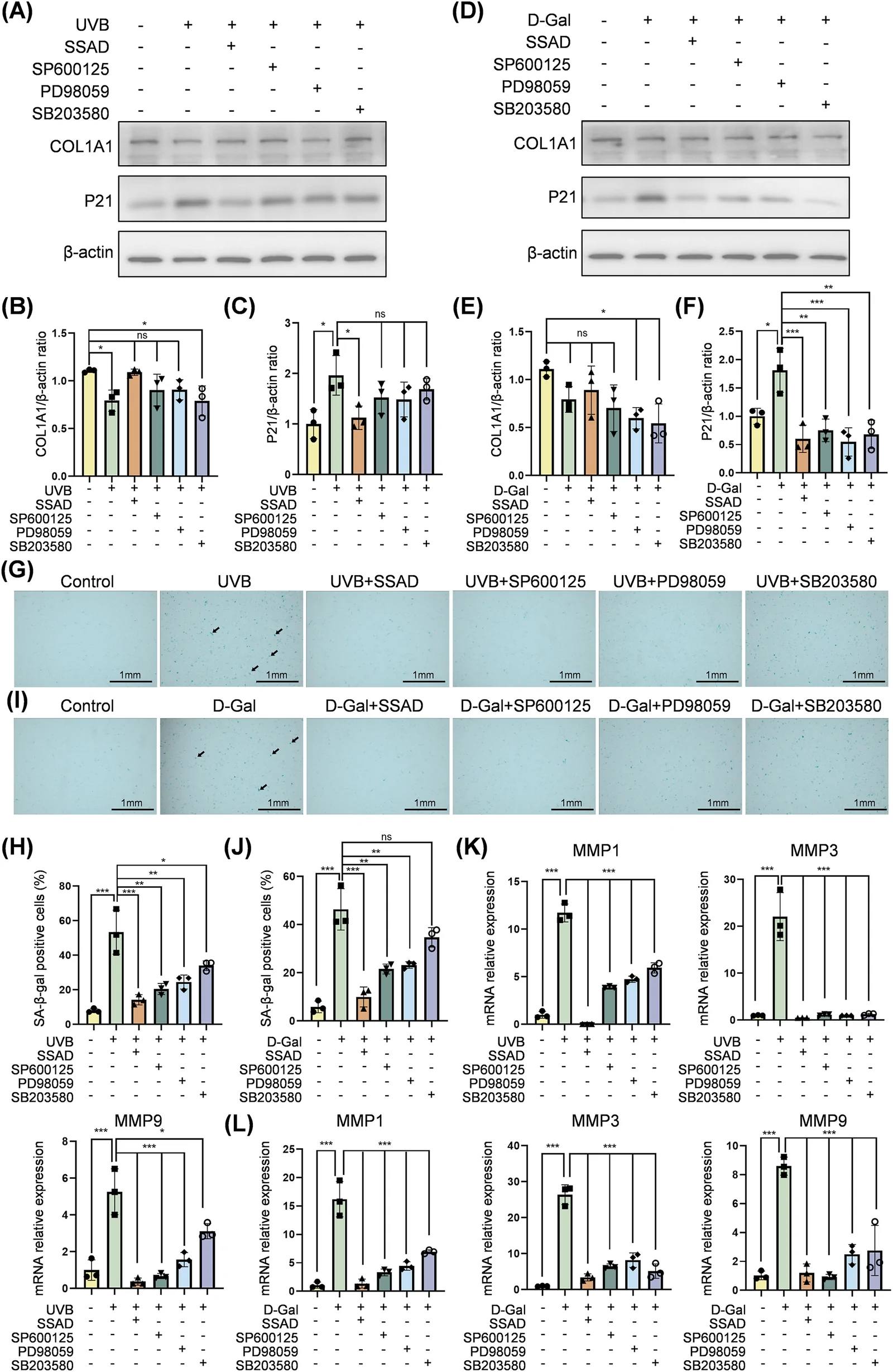
SSAD alleviates aging through the MAPK signaling pathway. **(A)** Western blot analysis showing the levels of P21 and collagen type I in UVB-treated cells following incubation with SSAD, PD98059, SP600125, or SB203580 (*n* = 3). **(B, C)** Quantitative analysis of the levels of P21 and collagen type I in the UVB model. **(D)** Western blot analysis showing the levels of P21 and collagen type I in D-Gal-treated cells following incubation with SSAD, PD98059, SP600125, or SB203580 (*n* = 3). **(E, F)** Quantitative analysis of the levels of P21 and collagen type I in the D-Gal model. **(G)** Representative SA-β-gal staining images of UVB-treated cells following incubation with SSAD, PD98059, SP600125, or SB203580 (arrows point to the positive cells) (*n* = 3). **(I)** Representative SA-β-gal staining images of D-Gal-treated cells following incubation with SSAD, PD98059, SP600125, or SB203580 (*n* = 3). **(H**, **J)** Quantitative analysis of the percentage of SA-β-gal-positive cells; qRT-PCR detection of the transcriptional levels of genes (MMP1, MMP3, MMP9) in the **(K)** UVB model and **(L)** D-Gal model (*n* = 3). ∗*p* < 0.05, ∗∗*p* < 0.001, ∗∗∗*p* < 0.0001.

### SSAD weakens UVB radiation-induced skin damage

To validate the therapeutic effects of SSAD, a UVB-induced skin aging mouse model was established (Fig. 7A). Following 7 days of UVB exposure, model mice exhibited significant skin hardening, pigmentation, and pronounced dermal vasodilation, whereas these changes were markedly milder in the UVB + SSAD group (Fig. 7B). Ultrasound imaging revealed that UVB irradiation decreased dermal collagen density (hyperechoic regions) and increased skin thickness, and these alterations were significantly ameliorated by SSAD treatment (Fig. 7C–F, G). Consistently, SSAD attenuated UVB-induced elevation of transepidermal water loss (TEWL), indicating preserved skin barrier function (Fig. 7H).

**Figure 7:**
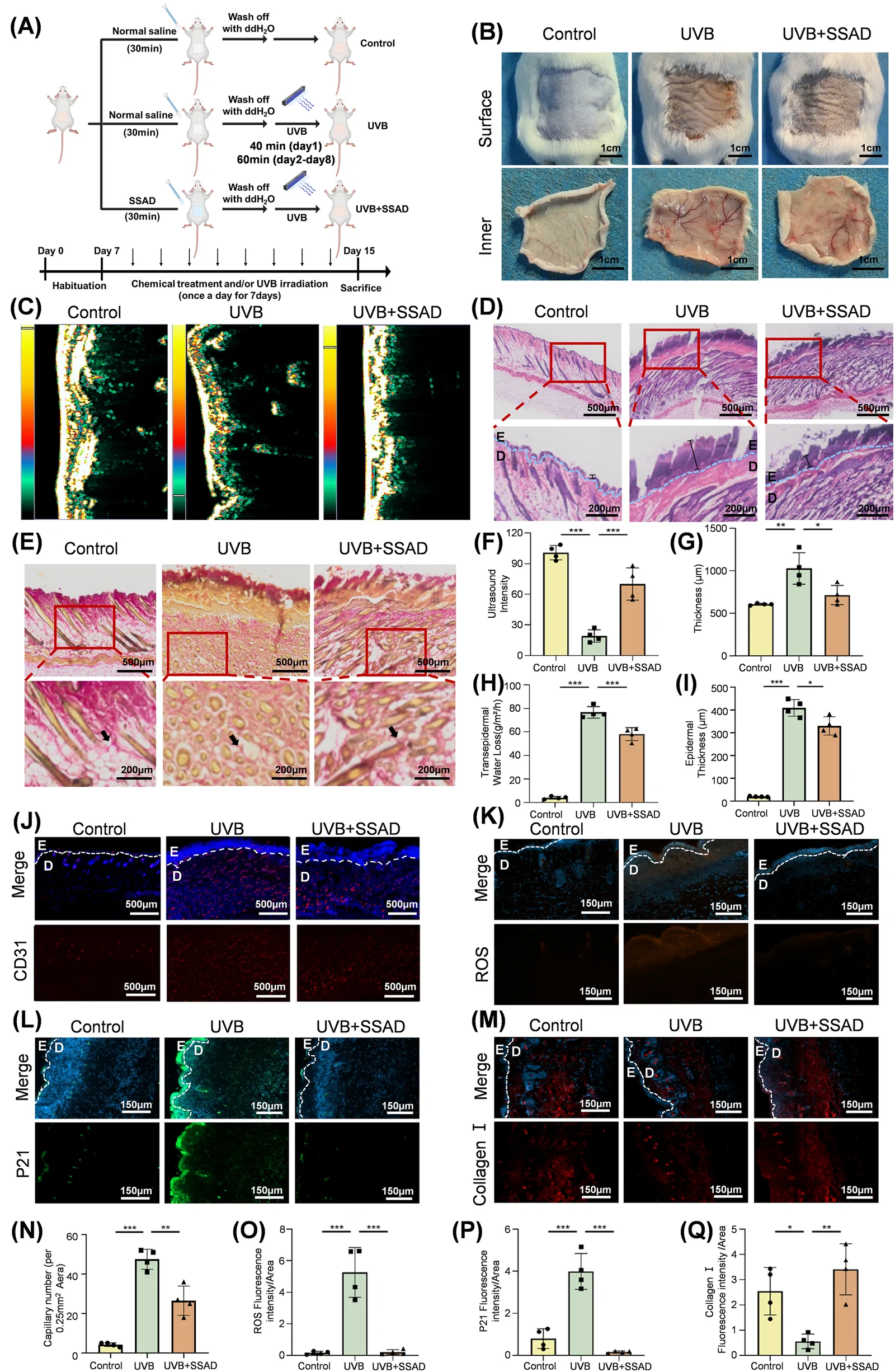
*In vivo* evaluation of SSAD aqueous extract in the photoaging model. **(A)** Schematic diagram of the establishment of the mouse skin UVB-photoaging model and the intervention procedure with SSAD aqueous extract. **(B)** Representative macroscopic images of depilated back skin tissue from mice after UVB irradiation. **(C)** Ultrasound measurement of skin collagen after UVB exposure (yellow and white areas represent collagen). **(D)** H&E staining of depilated skin tissue exposed to UVB radiation (E: epidermis; D: dermis), showing epidermal hyperplasia and alterations in dermal structure. **(E)** VG staining of depilated skin tissue exposed to UVB radiation. Arrows indicate collagen fibers (red), assessed for collagen density. **(F)** Quantitative analysis of skin collagen content after UVB exposure. **(G)** Quantitative analysis of skin thickness after UVB exposure. **(H)** Quantitative analysis of transepidermal water loss after UVB irradiation. **(I)** Quantitative analysis of epidermal thickness after UVB radiation. **(J)** IF detection of CD31 in back skin tissues for microvessel density evaluation; DAPI was used to visualize nuclei. **(K)** Red fluorescence showing ROS levels in the dorsal skin tissue, with blue fluorescence from DAPI indicating cell nuclei. **(L)** Green fluorescence showing P21 levels in the dorsal skin tissue, with DAPI indicating cell nuclei. **(M)** Red fluorescence showing type I collagen levels in the dorsal skin tissue, with DAPI indicating cell nuclei. **(N)** Quantitative analysis of vascular density in the dermis. **(O**–**Q)** Quantitative analysis of fluorescence intensity of ROS, P21 and collagen I in skin per unit area after UVB irradiation. ∗*p* < 0.05, ∗∗*p* < 0.001, ∗∗∗*p* < 0.0001.

Histopathological examination via H&E staining showed that SSAD significantly reduced UVB-induced epidermal hyperplasia and inflammatory infiltration (Fig. 7D–I). VG staining further revealed that SSAD mitigated UVB-induced collagen breakdown, as evidenced by deeper pink staining in the dermis compared to the UVB group (Fig. 7E). Immunofluorescence staining for CD31 demonstrated that SSAD treatment reduced the UVB-induced increase in dermal capillary density (Fig. 7J–N). To elucidate the underlying mechanisms, immunofluorescence staining was performed on skin sections. UVB irradiation markedly elevated ROS levels (red fluorescence) and P21 expression (green fluorescence) while decreasing collagen I fluorescence intensity (Fig. 7K–M). These alterations were significantly reversed by SSAD treatment (Fig. 7O–Q). Collectively, these results demonstrate that SSAD effectively mitigates UVB-induced skin aging and damage by preserving collagen density, maintaining barrier function, reducing oxidative stress, and suppressing cellular senescence.

## Discussion

Skin aging represents a complex, multifactorial biological process characterized by progressive functional decline, serving as a visible indicator of systemic aging.^43^^,^^44^ This process is mechanistically driven by established hallmarks of aging, including genomic instability, telomere attrition, cellular senescence, and chronic inflammation.^45^^,^^46^ It is noteworthy that ROS play a central role in both exogenous (e.g., photoaging) and endogenous aging pathways.^33^^,^^47^

ROS contribute to the aging process through multiple mechanisms: they not only directly damage mitochondrial DNA but also act as signaling molecules, activating key pathways involving Ras, p53, p21, and p16, thereby inducing cell cycle arrest and a senescent phenotype.^48^^,^^49^ Furthermore, ROS have been shown to inhibit autophagy, further impairing the cell's ability to clear damaged components and exacerbating functional decline associated with aging.^18^ At the molecular pathway level, ROS are key activators of the MAPK signaling pathway. Activated MAPK family members, including p38, ERK, and JNK, not only engage in complex crosstalk among themselves^50^, ^51^, ^52^ but also synergistically trigger the activation of the downstream NF-κB pathway. Specifically, JNK triggers the phosphorylation of the AP-1 transcription complex (including c-Jun and c-Fos).^53^^,^^54^ The activation of AP-1 significantly upregulates the expression of matrix metalloproteinases (MMPs), leading to the extensive degradation of collagen and elastic fibers within the ECM – the structural basis for skin wrinkling and laxity.^55^ In fact, a primary characteristic defining the phenotype of aged skin fibroblasts is the markedly enhanced activity of ECM-degrading MMPs.^56^^,^^57^ Therefore, prompt clearance of excess ROS is paramount for delaying skin aging.^58^

To counteract ROS-induced oxidative damage, the human body employs an intrinsic antioxidant defense system comprising key enzymes, such as superoxide dismutase (SOD), glutathione peroxidase (GPX), and catalase (CAT), which collectively neutralize ROS.^59^, ^60^, ^61^ However, the functionality of these endogenous antioxidant systems also declines during the aging process.^34^^,^^62^ The intake or application of exogenous antioxidants thus presents a viable strategy.^63^^,^^64^ Vitamin C and phenolic compounds, such as anthocyanins and resveratrol, are exceptional antioxidants with widespread applications in anti-aging.^65^^,^^66^ Research has shown that the natural antioxidant Rubus fruticosus B can protect the skin by inhibiting MAPK pathway activation via ROS clearance.^67^ Similarly, Arthrospira platensis nanoparticles can alleviate D-Gal-induced aging-related oxidative damage in the rat brain through their antioxidant activity and modulation of the MAPK signaling pathway.^31^

SSAD is a highly promising natural extract. Previous research has highlighted its potential for developing hemostatic powders, owing to its remarkable ability to control hemorrhage, promote wound healing, and facilitate tendon healing.^25^^,^^27^^,^^28^^,^^68^ Building on its well-established antioxidant properties,^28^ our investigation aimed to determine whether the aqueous extract of SSAD could effectively delay HSF senescence.

In this work, we initially assessed the antioxidant properties of the SSAD aqueous extract, confirming its potent free radical scavenging capacity.^32^ An intriguing finding was that although the SSAD group and the ASC group exhibited similar abilities to scavenge ROS, the SSAD group demonstrated superior improvements in other aging-related indicators, including cell migration capacity, senescence-associated β-Gal activity, formation of DNA damage marker γ-H2AX foci, and the expression profile of key genes. This suggests that the anti-aging effects of SSAD aqueous extract are not solely dependent on its antioxidant capacity and that additional mechanisms of action are likely involved. Subsequent investigation revealed that SSAD regulates the MAPK signaling pathway through a dual mechanism: direct inhibition of JNK phosphorylation and indirect suppression via ROS scavenging. Notably, a reciprocal regulatory relationship was identified in which pharmacological inhibition of MAPK also attenuated ROS levels, suggesting bidirectional crosstalk between the ROS and MAPK cascades. Furthermore, SSAD treatment effectively reversed UVB- or D-Gal-induced upregulation of p21 and MMPs and restored type I collagen expression, thereby recapitulating the effects of MAPK inhibitors. Collectively, these findings position SSAD as a multi-target natural agent that alleviates skin cell senescence by modulating the ROS-MAPK axis. While the precise molecular targets warrant further investigation, this study provides a mechanistic foundation for developing SSAD-based interventions against skin aging.

In summary, this work demonstrates that SSAD aqueous extract is a promising natural antioxidant agent. It effectively scavenges excess ROS induced by either D-Gal or UVB irradiation and subsequently delays HSF senescence by specifically inhibiting the phosphorylation of key MAPK pathway components, namely, JNK, ERK, and P38. These findings establish a mechanistic foundation for developing SSAD-based interventions against skin aging.

## CRediT authorship contribution statement

**Runqi Zhou:** Writing – original draft, Methodology, Investigation, Formal analysis, Data curation, Conceptualization. **Jing Yang:** Writing – original draft, Validation, Software, Investigation, Data curation. **Liuting Chen:** Writing – original draft, Methodology, Investigation, Formal analysis, Data curation, Conceptualization. **Siyu Peng:** Writing – original draft, Validation, Investigation, Data curation. **Xing Feng:** Investigation, Data curation. **Ximu Zhang:** Writing – review & editing, Supervision, Resources, Investigation, Funding acquisition, Data curation, Conceptualization. **Jinlin Song:** Writing – review & editing, Supervision, Resources, Investigation, Funding acquisition, Data curation, Conceptualization.

## Ethics declaration

All experimental protocols in this study were reviewed and approved by the Ethics Committee of Chongqing Medical University (Approval No. CQHS-REC-2024 (LSNo 005) and No. CQHS-REC-2023 (LSNo 188)). The acquisition of all human tissue samples was conducted in accordance with the principle of informed consent.

## Funding

This work was supported by the 10.13039/501100001809National Natural Science Foundation of China (No. U22A20314, 32070826), the 10.13039/501100012166National Key R&D Program of China (No. 2022YFC2504200), the Outstanding Youth Fund of Chongqing Natural Science Foundation (China) (No. CSTB2023NSCQ-JQX0006), the Science and Technology Research Project of Chongqing Education Commission (China) (No. KJQN202200471), and the CQMU Program for Youth Innovation in Future Medicine (China) (No. W0075).

## Conflict of interests

Jinlin Song is a member of *Genes & Diseases* Editorial Board. To minimize bias, he/she was excluded from all editorial decision-making related to the acceptance of this article for publication. The remaining authors declare that they have no known competing financial interests or personal relationships that could have appeared to influence the work reported in this paper.
