# Supplementary material for: Skin secretion of Andrias davidianus aqueous extract delays skin aging by suppressing the MAPK pathway
Source: Genes Dis. 2026 May 28;14(3):102255. doi: 10.1016/j.gendis.2026.102255 (PMC13770623; doi:10.1016/j.gendis.2026.102255)
Supplement: Supplementary file 1 [file mmc1.docx]

**The skin secretion of Andrias davidianus aqueous extract delays skin aging by suppressing the MAPK pathway**

Runqi Zhou^a,b,c,d,e^, Jing Yang^a,b,c,d,e^, Liuting Chen^a,b,c,d,e^, Siyu Peng^a,b,c,d,e^, Xing Feng^a,b,c,d^, Ximu Zhang^a,b,c,d*^, Jinlin Song^a,b,c,d*^

^a^ The Affiliated Stomatological Hospital of Chongqing Medical University, Chongqing 401147, China.

^b^ Chongqing Key Laboratory of Oral Diseases, Chongqing 401147, China.

^c^ Chongqing Municipal Key Laboratory of Oral Biomedical Engineering of Higher Education, Chongqing 401147, China.

^d^ Chongqing Municipal Health Commission Key Laboratory of Oral Biomedical Engineering, Chongqing 401147, China.

^*^Corresponding author. The Affiliated Stomatological Hospital of Chongqing Medical University, Chongqing 401147, China; Chongqing Key Laboratory of Oral Diseases, Chongqing 401147, China; Chongqing Municipal Key Laboratory of Oral Biomedical Engineering of Higher Education, Chongqing 401147, China; Chongqing Municipal Health Commission Key Laboratory of Oral Biomedical Engineering, Chongqing 401147, China.

E-mail addresses: songjinlin@hospital.cqmu.edu.cn (J. Song), zhangximu@hospital.cqmu.edu.cn (X. Zhang).

^e^ These authors contributed equally to this work.

**Supplementary Figures**

**Supplementary Figure 1** Preparation of SSAD aqueous extract.

**Supplementary Figure 2** Quantitative analysis of ascorbic acid (ASC) content in SSAD samples before and after dialysis by high-performance liquid chromatography (HPLC).

**Supplementary Figure 3** The cytotoxicity of SSAD on HSFs. (A) Cell viability of different concentrations of SSAD aqueous extract on HSF; (B) Cell viability of SSAD aqueous extract at different time points on HSF. ***P* < 0.01, ****P* < 0.001.

**Supplementary Figure 4** Effect of SSAD on MAPK signaling. (A) Quantitative RT-PCR detected the transcription levels of ERK, JNK, and p38 in the photoaging model (n=3); (B) Quantitative RT-PCR detected the transcription levels of ERK, JNK, and p38 in the D-Gal model (n=3). * *P* < 0.05, ** *P* < 0.001, *** *P* < 0.0001.

**Supplementary Figure 5** Images of the original WB bands.

**Supplementary Tables**

**Table S1．**qRT-PCR primer sequences.

| **Gene** | **Forward** | **Reverse** |
| --- | --- | --- |
| Human GAPDH | GCACCGTCAAGGCTGAGAAC | TGGTGAAGACGCCAGTGGA |
| Human SOD1 | TGAGTTTGGAGATAATACAGCAGGC | GTCAGCAGTCACATTGCCCA |
| Human GPX1 | CCGCTTCCAGACCATTGACAT | ATCAGGTGTTCCTCCCTCGTA |
| Human P16 | CTCGTGCTGATGCTACTGAGGA | GGTCGGCGCAGTTGGGCTCC |
| Human MMP3 | CACTCACAGACCTGACTCGGTT | AAGCAGGATCACAGTTGGCTGG |
| Human MMP1 | ATGAAGCAGCCCAGATGTGGAG | TGGTCCACATCTGCTCTTGGCA |
| Human MMP9 | GCCACTACTGTGCCTTTGAGTC | CCCTCAGAGAATCGCCAGTACT |
| Human IL-1β | CCACAGACCTTCCAGGAGAATG | GTGCAGTTCAGTGATCGTACAGG |
| Human TNF-α | CTCTTCTGCCTGCTGCACTTTG | ATGGGCTACAGGCTTGTCACTC |
| Human COLA1 | CTGGACAACCTGGTCAAGATGG | CCTTTGTAACCTGGTAGTCCTCG |
| Human JNK | TGAGAAACTCTTCCCTGATGTCC | ACAAATCCCTTGCCTGACTGG |
| Human ERK | GGCTGTTCCCAAATGCTGAC | AACTTGAATGGTGCTTCGGC |
| Human P38 | CTGTTGGACGTTTTTACACCTGC | AGACCTCGGAGAATTTGGTAGA |

Table S2 Antibodies used for WB and IF.

| **Antibody** | **Company** | **Dilution** |
| --- | --- | --- |
| p38 MAPK (D13E1) XP® Rabbit mAb #8690 | Cell Signaling Technology | 1:1000 |
| Phospho-p38 MAPK (Thr180/Tyr182) (D3F9) XP® Rabbit mAb #4511 | Cell Signaling Technology | 1:1000 |
| p44/42 MAPK (Erk1/2) (137F5) Rabbit mAb #4695 | Cell Signaling Technology | 1:1000 |
| Phospho-p44/42 MAPK (Erk1/2) (Thr202/Tyr204) (197G2) Rabbit mAb #4377 | Cell Signaling Technology | 1:1000 |
| SAPK/JNK Antibody #9252 | Cell Signaling Technology | 1:1000 |
| Phospho-SAPK/JNK (Thr183/Tyr185) (81E11) Rabbit mAb #4668 | Cell Signaling Technology | 1:1000 |
| Phospho-Histone H2A.X (Ser139) (20E3) Rabbit mAb #9718 | Cell Signaling Technology | 1:200 |
| CD31 (PECAM-1) (89C2) Mouse mAb #3528 | Cell Signaling Technology | 1:200 |
| Beta Actin Monoclonal antibody(2D4H5)  P21 Polyclonal antibody (10355-1-AP)  Collagen Type I Monoclonal antibody (66761-1-Ig) | Proteintech  Proteintech  Proteintech | 1:50000  1:1000  1:1000 |

**Table S3．**a five-point standard curve of ASC standard solution
